# Oxytocin neurons enable social transmission of maternal behaviour

**DOI:** 10.1038/s41586-021-03814-7

**Published:** 2021-08-11

**Authors:** Ioana Carcea, Naomi López Caraballo, Bianca J. Marlin, Rumi Ooyama, Justin S. Riceberg, Joyce M. Mendoza Navarro, Maya Opendak, Veronica E. Diaz, Luisa Schuster, Maria I. Alvarado Torres, Harper Lethin, Daniel Ramos, Jessica Minder, Sebastian L. Mendoza, Chloe J. Bair-Marshall, Grace H. Samadjopoulos, Shizu Hidema, Annegret Falkner, Dayu Lin, Adam Mar, Youssef Z. Wadghiri, Katsuhiko Nishimori, Takefumi Kikusui, Kazutaka Mogi, Regina M. Sullivan, Robert C. Froemke

**Affiliations:** 1 Skirball Institute for Biomolecular Medicine, New York University School of Medicine, New York, NY USA; 2 Neuroscience Institute, New York University School of Medicine, New York, NY USA; 3 Department of Otolaryngology, New York University School of Medicine, New York, NY USA; 4 Department of Neuroscience and Physiology, New York University School of Medicine, New York, NY USA; 5 Department of Pharmacology, Physiology and Neuroscience, Brain Health Institute, Rutgers, The State University of New Jersey, New Jersey, USA; 6 Department of Animal Science and Biotechnology, Azabu University, Sagamihara, Kanagawa Japan; 7 Department of Neuroscience, Mount Sinai School of Medicine, New York, NY USA; 8 Emotional Brain Institute, Nathan Kline Institute for Psychiatric Research, Orangeburg, NY USA; 9 Department of Child and Adolescent Psychiatry, New York University School of Medicine, New York, NY USA; 10 Center for Advanced Imaging Innovation and Research, Bernard and Irene Schwartz Center for Biomedical Imaging, Department of Radiology, New York University School of Medicine, New York, NY USA; 11 Department of Obesity and Internal Inflammation, Fukushima Medical University, Fukushima, Japan; 12 Department of Psychiatry, New York University School of Medicine, New York, NY USA; 13 Present Address: Department of Psychology, Zuckerman Neuroscience Institute, Columbia University, New York, NY USA; 14 Present Address: Princeton Neuroscience Institute, Princeton, NJ USA

**Keywords:** Neural circuits, Social behaviour

## Abstract

Maternal care, including by non-biological parents, is important for offspring survival^1–8^. Oxytocin^1,2,9–15^, which is released by the hypothalamic paraventricular nucleus (PVN), is a critical maternal hormone. In mice, oxytocin enables neuroplasticity in the auditory cortex for maternal recognition of pup distress^15^. However, it is unclear how initial parental experience promotes hypothalamic signalling and cortical plasticity for reliable maternal care. Here we continuously monitored the behaviour of female virgin mice co-housed with an experienced mother and litter. This documentary approach was synchronized with neural recordings from the virgin PVN, including oxytocin neurons. These cells were activated as virgins were enlisted in maternal care by experienced mothers, who shepherded virgins into the nest and demonstrated pup retrieval. Virgins visually observed maternal retrieval, which activated PVN oxytocin neurons and promoted alloparenting. Thus rodents can acquire maternal behaviour by social transmission, providing a mechanism for adapting the brains of adult caregivers to infant needs via endogenous oxytocin.

## Abstract (web summary)

Behavioural studies and neural recordings in mice show that virgin mice can acquire maternal behaviour through an oxytocin-dependent mechanism.

## Introduction

Social interactions, such as pair bond formation and child rearing, are fundamental aspects of animal and human behaviour^1–4^. Parental care is especially important, and is therefore believed to be at least partially innate or induced after mating. However, maternal behaviour can also be acquired from experience. In primates, including humans, non-biological parents can learn to care for children after instruction or observation of experienced caretakers and infants^1–8^. However, it is unclear whether alloparenting can be learned from experience in other species, and what neural mechanisms might underlie maternal learning.

The evolutionarily ancient hormone oxytocin is an important molecular signal for maternal behaviour^1,2,9,10^. In mammals, oxytocin release from the hypothalamus is associated with childbirth and lactation^10,11^. Oxytocin also acts in the brain to increase the salience of social information and enable alloparenting in mice^1,9,12–14^. Pup-naive virgin female mice initially ignore neonates and ultrasonic distress calls emitted by isolated pups^15^. However, within days of co-housing with experienced mothers (dams) and litters, most virgin females begin expressing alloparenting behaviours, including retrieving pups to the nest. Oxytocin accelerates the onset of pup retrieval, promoting plasticity in virgin auditory cortex for recognizing pup calls^15^. However, little is known about when PVN neurons in nulliparous, non-lactating adults are activated to promote alloparenting behaviour.

## Continuous behaviour monitoring over days

**Fig. 1:**
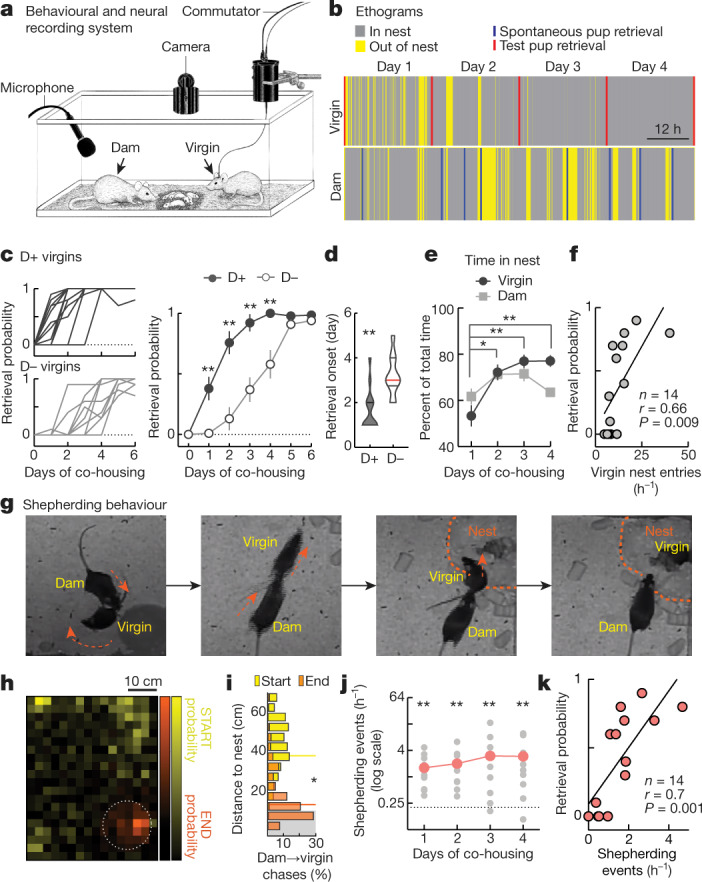
Dams shepherd virgins to nest. **a**, System for continuous monitoring of behaviour and neural activity. **b**, Ethogram showing activities of co-housed dam and virgin mice over a period of four days. **c**, Co-housing with dam and pups led to earlier retrieval by virgins. Left, individual retrieval rates. Right, mean retrieval probability. **d**, Day of retrieval onset was earlier in D+ virgins. **e**, Time in nest for dams and co-housed virgin mice. **f**, Correlation between virgin nest entry during day 1 and virgin retrieval at the end of day 1. **g**, Illustration of shepherding behaviour; arrows, movement direction; dashed circle, nest area. **h**, Probabilities of dam starting (yellow) and ending (orange) chasing of a co-housed virgin, relative to nest position (circle indicates the nest, radius approximately 10 cm). **i**, Distances from nest of dam→virgin chases. **j**, Frequency of shepherding events (grey, individual dyads; red, daily averages across cages) was more frequent than dam→virgin chases in the absence of pups (0.2, indicated by dashed line). **k**, Correlation between shepherding during day 1 and virgin retrieval at day 1 end. Data are mean ± s.e.m.; *P < 0.05, **P < 0.01.

**Extended Data Fig. 1:**
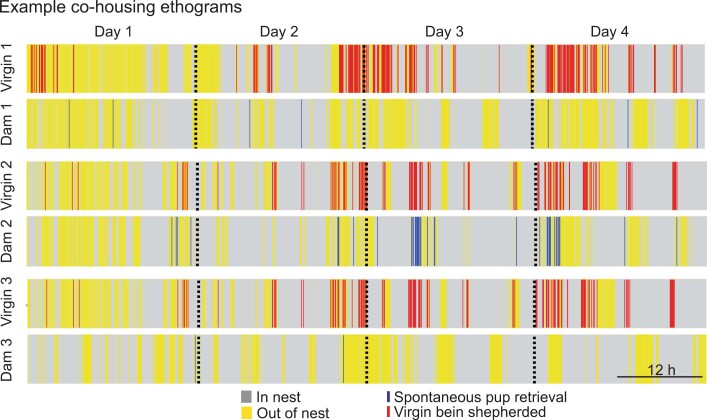
Behavioural ethograms from 3 pairs of animals. Ethograms were constructed from visual analysis of continuous four-day videography by two to four independent observers. Dashed vertical lines indicate end of each day.

Emergence of pup retrieval in co-housed virgin females provides an opportunity to monitor neural activity during interactions with adults and infants. To examine the behavioural events and neural activity patterns that lead to maternal behaviour in virgin female mice, we built a system for days-long combined behavioural and neural activity monitoring in cages of mother, litter and co-housed virgin female mice (Fig. 1a). This system consists of an overhead camera imaging homecages with visible (daytime) and infrared light (nighttime), synchronized with ultrasonic audio recordings and amplifiers for neural recordings. We constructed ethograms^16^ and quantified the frequency and duration of specific behaviours (for example, spontaneous pup retrieval and time in nest; Fig. 1b, Extended Data Fig. 1, Supplementary Videos 1, 2). We examined daily pup retrieval offline to determine when virgins began reliably responding to infant calls (Fig. 1b, c).

**Extended Data Fig. 2:**
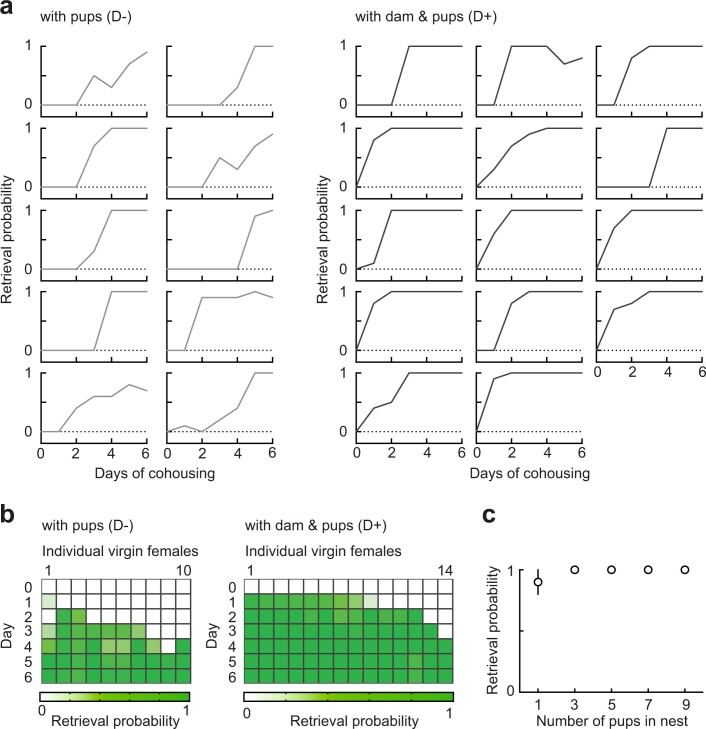
Pup retrieval over animals. **a**, Retrieval probabilities of all virgins co-housed with just pups (D-, left) or with experienced dam and pups (D+, right). **b**, Summary of virign retrieval probabilities over days, color indicates retrieval success each day. **c**, Dam retrieval probability uniformly high across dams with different litter sizes (N=3 dams, p>0.9, Kruskal-Wallis test). Error bars, SEM.

We found that the presence of the mother accelerated the onset of alloparenting in co-housed virgins. Virgins co-housed with dams and litters (D+ virgins, *n* = 14) began reliably retrieving (in more than 1 out of 10 trials) on day two of co-housing, earlier than virgins co-housed with pups but without dams (D− virgins, *n* = 10) (Fig. 1c, d, Extended Data Fig. 2a, b, Supplementary Videos 3,4; *P* < 0.0001). Pup retrieval was not initially performed by virgins but was reliably performed by dams regardless of litter size (Extended Data Fig. 2c). Virgins also began to spend time in the nest with pups (Fig. 1b, e; *P* < 0.0001), and the more virgins entered the nest, the more likely it was that retrieval occurred on day 1 (Fig. 1f; *r* = 0.66, *P* = 0.009).

## PVN activity when dams shepherd virgins

**Extended Data Fig. 3:**
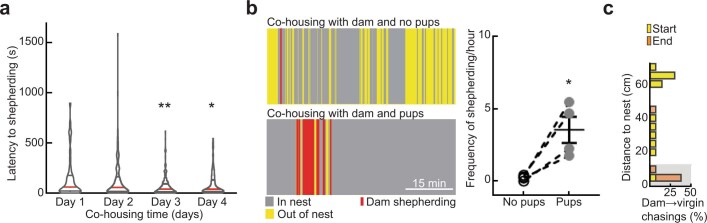
Shepherding behaviour. **a**, Average latency for dams to shepherd virgin to the nest (time 0 is virgin nest exit). Latency decreased over days of co-housing (day 1: 155.9±34 s; day 2: 136.1±17.94 s; day 3: 75.7±5.5 s; day 4: 93.9±8.5 s; n=676 events from N=9 dam-virgin pairs, p<0.0001, one-way ANOVA, Holm-Sidak’s multiple-comparison test). Red lines, medians. Error bars, SEM. **b**, Left, ethograms from two separate virgins, one co-housed just with dam without pups (top), the other co-housed with dam and pups (bottom). Note differences between time in nest and amount of shepherding when pups were present. Right, **s**ummary of shepherding events per hour when pups were removed vs when pups were present (‘No pups’: 0.2±0.1 events/hour; ‘Pups’: 3.5±0.9 events/hour, N=4, p=0.03, Student’s two-tailed paired t-test). Error bars, SEM. **c**, Locations of start (yellow) and end (orange) positions of dam→virgin chasing events without pups in the cage (N=4 dyads), relative to nest center (nest radius was ~10 cm, gray). 46.1±10.4% of dam→virgin chases ended in the nest, indicating that the frequency of these chases was significantly reduced but the direction of the virgins was comparable with or without pups (all chasing events ending in nest, p=0.9, two-sided Fisher’s exact test).

To determine how dams influenced virgin alloparenting, we analysed four days of video per cage and observed some unusual interactions between dams and virgins. First, mothers attempted to keep virgins within the nest with pups. If the virgin left the nest, the mother would escort or ‘shepherd’ her back (Fig. 1g–j, Supplementary Videos 5–7). Shepherding behaviour emerged over hours to days (Extended Data Fig. 3a) and seemed different from maternal aggression; rather than preventing contact with pups, dams encouraged virgins to enter and remain in the nest. Shepherding was unlikely to be dams ‘retrieving’ virgins as this rarely occurred without pups (Extended Data Fig. 3b, c). Shepherding occurred hundreds of times during co-housing (Fig. 1j, Extended Data Fig. 1a), and its frequency correlated with virgin retrieval at day 1 (Fig. 1k; *r* = 0.75, *P* = 0.001).

**Fig. 2:**
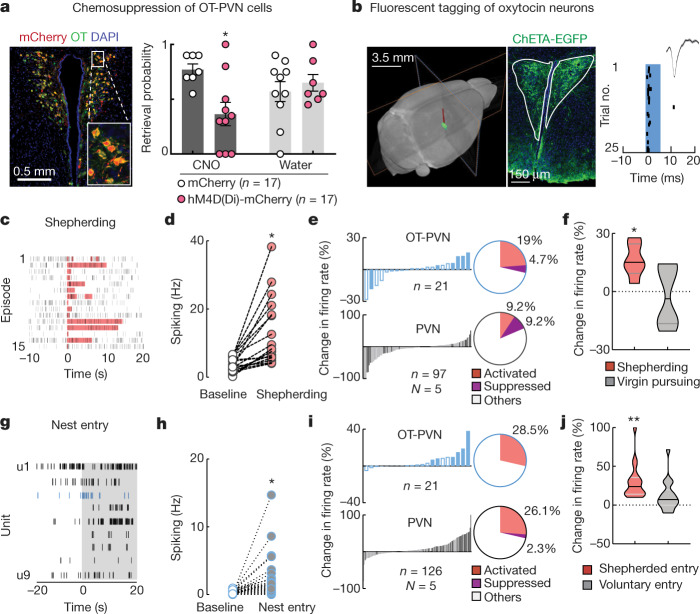
Shepherding and nest entry activate virgin PVN/OT-PVN neurons. **a**, Left, DREADDi–mCherry in OT-PVN cells. Right, CNO treatment during initial co-housing reduced retrieval in DREADDi-expressing mice. **b**, Optically tagged OT-PVN neurons in vivo. Left, µCT image used to localize electrodes. Middle, ChETA–eGFP expression in OT-PVN neurons. Right, raster plot of photo-tagged OT-PVN neuron; inset, waveform; blue bar, light pulse. **c**, **d**, Raster plot (**c**) and before–after plot (**d**) of PVN single unit from co-housed virgin during shepherding (shading). **e**, Change in spiking rate during shepherding for individual units. Filled bars indicate significant changes in spiking rate. **f**, Units with significant rate changes during shepherding (red) fired less during virgin→dam chases (grey). **g**, Simultaneously recorded PVN cells including OT-PVN cell (u3) during nest entry event. Units are numbered u1–u9. **h**, u3 spiking during nest entry (*n* = 140 events). **i**, Firing rate during nest entry. **j**, Spiking rate when virgin entered nest after shepherding (red) or voluntarily (grey). Data are mean ± s.e.m.; *P < 0.05, **P < 0.01.

**Extended Data Fig. 4:**
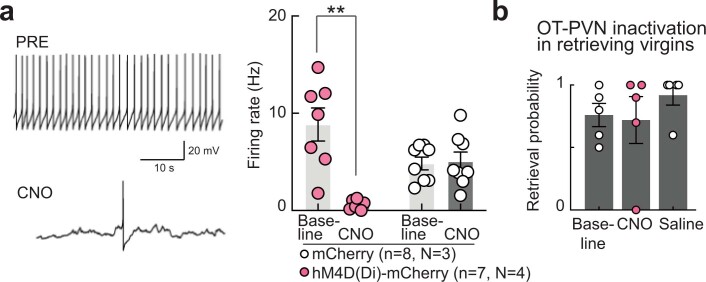
Controls for DREADDi-based impairment of oxytocin system during acquisition of pup retrieval behaviour. **a**, Recordings in female mouse PVN brain slices. Left, current-clamp whole-cell recording from OT-PVN neuron expressing DREADDi-mCherry before (top) and after (bottom) CNO bath administration. Right, summary of chemogenetic suppression of OT-PVN neuronal firing in brain slices (‘hM4D(Di)-mCherry’ baseline firing rate before CNO: 8.8±1.7 Hz, after CNO: 0.6±0.1 Hz, n=7 cells from N=4 mice; ‘mCherry’ baseline firing rate before CNO: 4.8±0.6 Hz, after CNO: 5.0±0.9 Hz, n=8 cells from N=3 mice; two-way ANOVA with Sidak’s correction for multiple comparisons, p=0.0002 between baseline and CNO for hM4D(Di)-mCherry, p=0.9 between baseline and CNO for mCherry). Error bars, SEM. **b**, CNO in retrieving females that expressed DREADDi did not affect retrieval rates (baseline retrieval: 76.0±9.2%, N=5; CNO: 72.0±18.8%, N=5; saline: 92.0±8.0%, N=5; repeated measures ANOVA, p=0.332). Error bars, SEM.

We hypothesized that co-housing led to virgin alloparenting via oxytocin. We used designer receptors exclusively activated by designer drugs coupled to inhibitory G-protein receptors (DREADDi) to silence PVN oxytocin neurons (OT-PVN cells; Extended Data Fig. 4a), and found that retrieval onset in naive co-housed virgins was delayed (Fig. 2a). However, silencing of OT-PVN cells had little effect after virgins began retrieving (Extended Data Fig. 4b), similar to primiparous dams^17^.

**Extended Data Fig. 5:**
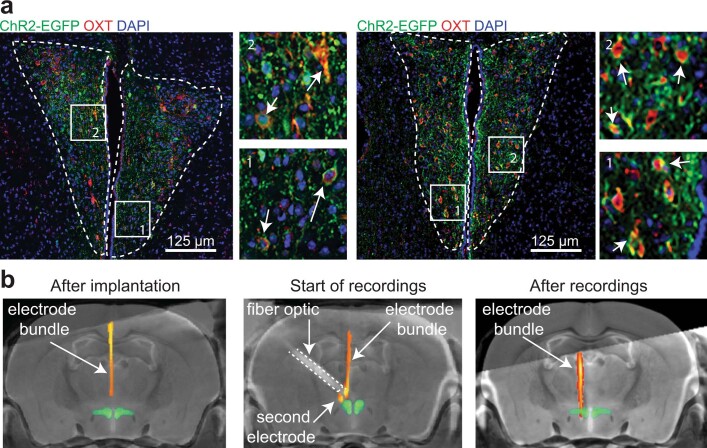
Confirmation of opsin expression and targeting for optically tagged OT-PVN recordings. **a**, Example immunostained tissue sections of PVN showing co-labeling of oxytocin peptide (red) and ChETA channelrhodopsin 2 conjugated to EGFP (green) together with DAPI labeling for cell density (blue). Insets show 2x magnified images to highlight the co-localization of red and green staining (arrows) in PVN cells. 84.8±7.2% of cells (N=6 animals) expressing ChETA also expressed oxytocin peptide. **b**, Verification of electrode locations in PVN with combined MRI/µCT. µCT imaging was performed after implantation and subsequently co-registered with a mouse MRI atlas to localize electrode bundles within PVN, as well as the separate fibre optic implanted for optogenetic identification of oxytocin neurons. Electrodes were then lowered at the end of each day of co-housing. Yellow/orange, µCT imaging of electrodes in situ. Images shown are from different animals.

**Extended Data Fig. 6:**
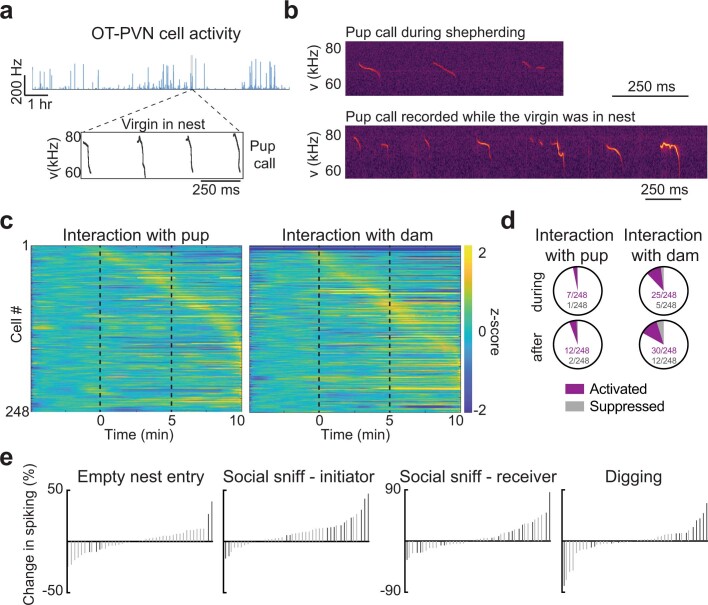
PVN neurons in adult female virgin mice responded more robustly during interactions with dams than with pups. **a**, Example of OT-PVN neuron instantaneous firing (top), synchronized with homecage behaviour (gray bar marks a nest entry episode), and with pup vocalizations (bottom), illustrates the different data streams recorded with our system. **b**, Example spectrograms of pup vocalizations and distress calls in home cage during shepherding and nest entry. **c**, Normalized (z-scored) spiking for 248 PVN neurons recorded during 5-minute interaction periods with pup (left) or a dam (right). Recordings sorted by peak activity. **d**, Proportion of activated (purple; z-score > 1.645, i.e. p < 0.05) and suppressed (gray, z-score < −1.645) PVN cells. During the 5-minute interaction periods, interactions with dam activated more virgin PVN cells than interactions with pups (with dam: 25/248, with pup: 7/248, p=0.001, two-sided Fisher’s exact test). After dam interactions, PVN cells continued to fire more than after interactions with pup (with dam: 30/248, with pup: 12/248, p=0.005). **e**, Social sniffing between the virgin and dam activated PVN neurons (9/43 neurons significantly activated while the virgin was sniffed, p=0.002; 11/43 neurons activated while the virgin sniffed the other adult, p=0.0005). A similar fraction of PVN neurons was activated during digging (8/43, p=0.005). Only a few PVN neurons were activated by entering an empty nest without pups (2/43, p=0.4). Dark bars, significantly modulated neurons. The proportion of PVN neurons activated during empty nest entry was smaller than the proportion of PVN neurons activated while being sniffed (two-sided Fisher’s exact test, p=0.04), during sniffing (p=0.01), and after entering a nest with pups (p=0.001). Fraction of cells responding during empty nest entry was also smaller than the proportion of OT-PVN neurons activated during shepherding (0.03), dam retrieval (p=0.01), and after entering a nest with pups (p=0.01).

**Extended Data Fig. 7:**
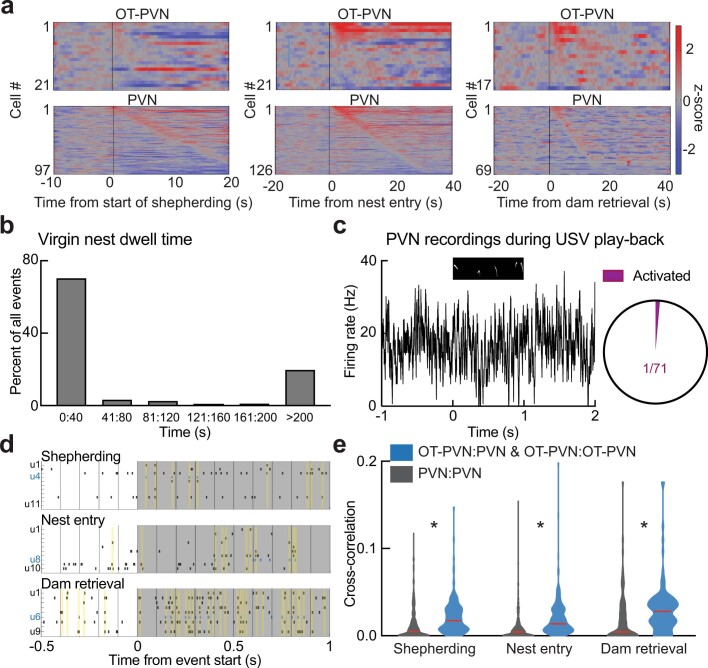
PVN unit activity related to specific interactions with dams or pups. **a**, Patterns of OT-PVN (top) and PVN (bottom) cell activity during shepherding (left; 5/21 OT-PVN neurons, Fisher’s exact test, p=0.02; 9/97 PVN neurons, p=0.001), nest entry (middle; 6/21 OT-PVN neurons, p=0.01; 33/126 PVN neurons, p=0.0001; shepherding vs voluntary entry, p=0.005, Wilcoxon signed-rank test) and dam retrieval (right; 5/17 OT-PVN neurons, p=0.02; 10/69 PVN neurons, p=0.0007). Recordings were aligned to start of interaction, and sorted by peak activity. **b**, Distribution of durations of time in nest for virgins after entry events. ~70% of all nest dwell times are ≤40 s. **c**, Left, example PSTH for PVN unit during play-back of pup distress calls (sonogram on top). Right, summary of PVN activity during pup call presentation. Almost all cells in virgin PVN were unresponsive, with only 1/71 PVN units having significant responses to pup vocalizations. **d**, Examples of simultaneously recorded unit spike trains aligned to onset of individual shepherding, nest entry, and dam retrieval events during co-housing. Blue, OT-PVN units. Yellow, periods of coincidental activity within 10 ms. **e**, Summary of average zero-lag pair correlations for each pair of simultaneously recorded units during each co-housing behaviour. Neuronal pairs including OT-PVN neurons (OT-PVN:OT-PVN and OT-PVN:PVN pairs) were more strongly correlated than PVN:PVN pairs (shepherding, n=530 pairs, p<0.0001; nest entry, n=776 pairs, p<0.0001; dam retrieval, n=278, p<0.0001; ANOVA and Tukey’s multiple-comparison test). Error bars, SEM.

We investigated the interactions that activate virgin PVN and OT-PVN cells. We performed single-unit recordings from virgin PVN during co-housing, including optically identified OT-PVN units expressing channelrhodopsin 2 (Fig. 2b, Extended Data Figs. 5a, 6a, b; *n* = 17 virgins, 541 PVN units, 21 OT-PVN units), localizing electrodes with co-registered micro-computed tomography (μCT) and MRI (Fig. 2b, Extended Data Fig. 5b). Some units were recorded offline during retrieval testing or other dam–pup interactions (Extended Data Fig. 6c–e). Virgin PVN and OT-PVN units were activated by shepherding or initial nest entry, with less activation from chasing dams, entry into an empty nest or pup calls (Fig. 2c–j, Extended Data Figs. 6c–e, 7a–c).

## Social transmission of maternal behaviour

**Fig. 3:**
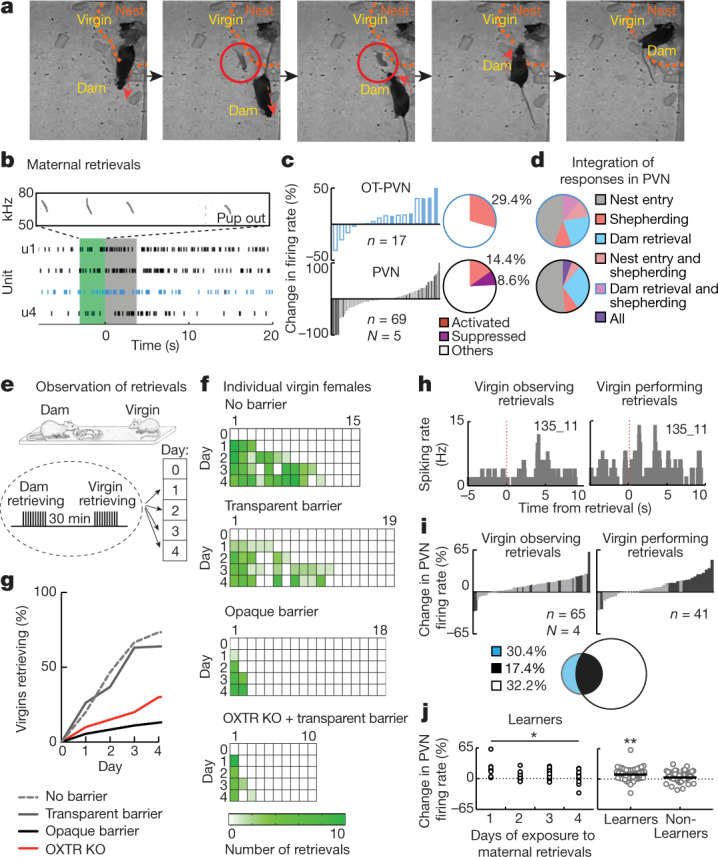
Observation of pup retrieval. **a**, Illustration of spontaneous retrieval by dam; arrows, movement direction; dashed lines, nest area; red circle, dropped pup. **b**, Activities of simultaneously recorded virgin PVN cells including OT-PVN cell (blue) during spontaneous retrieval episode by dam. Green, pup dropped; grey, pup retrieval. Top, frequency of emitted sounds. **c**, Spiking rate change in virgins during spontaneous retrievals by dams. **d**, Heterogeneous responses (*n* = 35 (PVN), *n* = 9 (OT-PVN) units) during nest entry, shepherding, and maternal retrieval. Top, OT-PVN; bottom, PVN. **e**, Pup retrieval by D− virgins. **f**, Retrieval by D− virgins retrieval was higher after exposure to dam retrieval with or without a transparent barrier compared with an opaque barrier or with OXTR-KO virgins. **g**, Cumulative distributions (Kaplan–Meier) for retrieval onset. **h**, Example virgin PVN unit responding to both observation of maternal retrieval and self-performance of retrieval (time 0, pup retrieved). **i**, Change in virgin PVN firing rate for units recorded during observation of dam retrievals (blue) and self-performance (white). **j**, PVN unit activity increased during observation in learners (*n* = 65 units, *N* = 3 virgins) but not non-learners (*n* = 103 units, *N* = 5 virgins). *P < 0.05, **P < 0.01.

**Extended Data Fig. 8:**
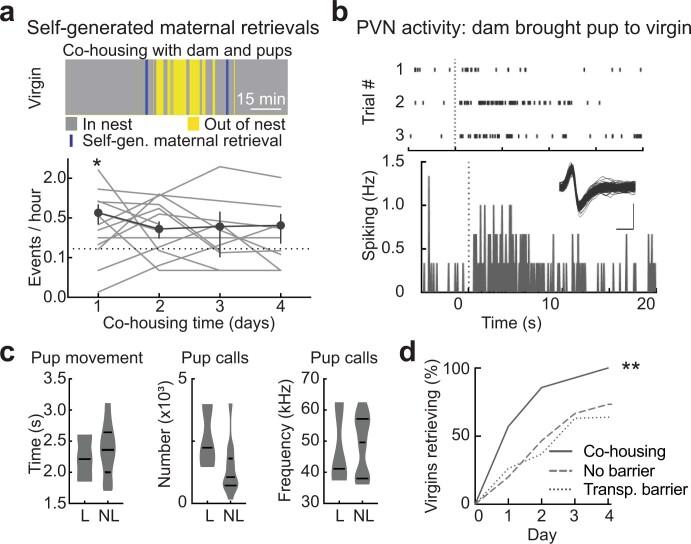
Many virgins seem to learn retrieval from maternal demonstration. **a**, Frequency of self-generated retrievals by dams. Top, example ethogram depicting two separate spontaneous retrievals by the dam (blue). Bottom, summary of individual dam spontaneous hourly pup retrieval rate per day (day one: 0.6±0.2 events/hour, day two: 0.3±0.1 events/hour, day three: 0.4±0.2 events/hour, day four: 0.4±0.2 events/hour, N=14). Lines, individual dam behaviour. Filled circles, daily average across animals. Dashed line, average rate of spontaneous retrieval for singly housed dams and litters without other adults (0.2±0.1 events/hour, N=5). Error bars, SEM. **b**, Virgin PVN responses when mother brought pups to virgin. For these animals, three separate pup delivery episodes occurred in a twenty-minute period after the second day of co-housing. On each of those occurrences, this PVN unit recorded in the virgin responded with increased spiking for several seconds until the dam returned to retrieve the pup herself. Top, raster of each of these three trials. Bottom, summary PSTH. Inset, spike waveforms; scale: 0.4 ms, 0.5 mV. **c**, Pup activity for non-co-housed virgins tested across transparent barrier, separated by virgins that began retrieving after observation (‘L’) and virgins that did not begin retrieving after observation (‘NL’). Left, pup movement (p=0.64, Student’s unpaired two-tailed t-test, N=16 animals: 3 learners, 13 non-learners). Middle, number of pup vocalizations (p=0.07). Right, pup call mean frequency (p=0.77). Error bars, SEM. **d**, Onset of retrieval behaviour was faster in co-housed virgins relative to non-co-housed virgins exposed to retrievals behind transparent barrier or with no barrier (Mantel-Cox test, p=0.004, N=48 mice total). **, p<0.01.

We also found that virgin alloparenting and PVN activity increased after spontaneous pup retrieval by dams. About once every two hours, dams dropped and then retrieved pups, sometimes bringing pups to the virgin (Fig. 3a, Extended Data Figs. 1, 8a, b, Supplementary Videos 1, 8–10). Remarkably, retrieval by dams evoked responses in virgin PVN (Fig. 3b, c), although the virgin was not directly involved. Some PVN and OT-PVN units responded to both shepherding and dam retrieval, but population responses were heterogeneous (Fig. 3d). We analysed cross-correlations across simultaneously recorded units, and found that pairs containing OT-PVN cells were more correlated during shepherding, nest entry and dam retrieval (Extended Data Fig. 7d, e). These data suggest that diverse experiences activate oxytocin neurons, ensuring that new parents become attuned to infant needs.

Additionally, these results indicate that virgins watch dams engage in maternal care, accelerating alloparenting via social transmission to observing virgins. We hypothesized that alloparenting could also be enhanced in non-co-housed virgins observing pup retrieval by dams. In separate non-co-housed virgins, for four consecutive days we performed ten retrieval tests with the dam (forty dam retrievals total) with the virgin present. Retrieval was assessed in virgins approximately 30 min later. Onset of retrieval in virgins increased after observed dam retrievals (Fig. 3e–g, No barrier: 11 out of 15 virgins retrieving after 40 maternal demonstrations; *P* = 0.0002, Fisher’s exact test), but independently of pup behaviours (Extended Data Fig. 8c).

We tested whether this transmission of retrieval behaviour involved physical contact by inserting a transparent barried between other observer pup-naive virgins and the retrieving dams. Most virgins behind a transparent barrier learned to retrieve (Fig. 3f, g, Supplementary Video 11; transparent: 12 out of 19 virgins retrieving, *P* < 0.0001). Visual input was required, as most non-co-housed virgins behind an opaque barrier did not begin retrieving (Fig. 3f, g, Supplementary Video 12; opaque: 2 out of 18 virgins retrieving, *P* = 0.2). Oxytocin signalling was also important, as oxytocin receptor (OXTR)-knockout (KO) virgins observing maternal retrievals through a transparent barrier did not begin retrieving (Fig. 3f, g, OXTR-KO: 3 out of 10 virgins retrieving, *P* = 0.1). The retrieval-learning rate of non-co-housed virgins was slower than that if co-housed virgins (Fig. 1c, Extended Data Fig. 8d), perhaps owing to heterogeneity of PVN and OT-PVN responses to maternal interactions, such that the variety of experiences occurring during co-housing more effectively activates the central oxytocin system.

**Extended Data Fig. 9:**
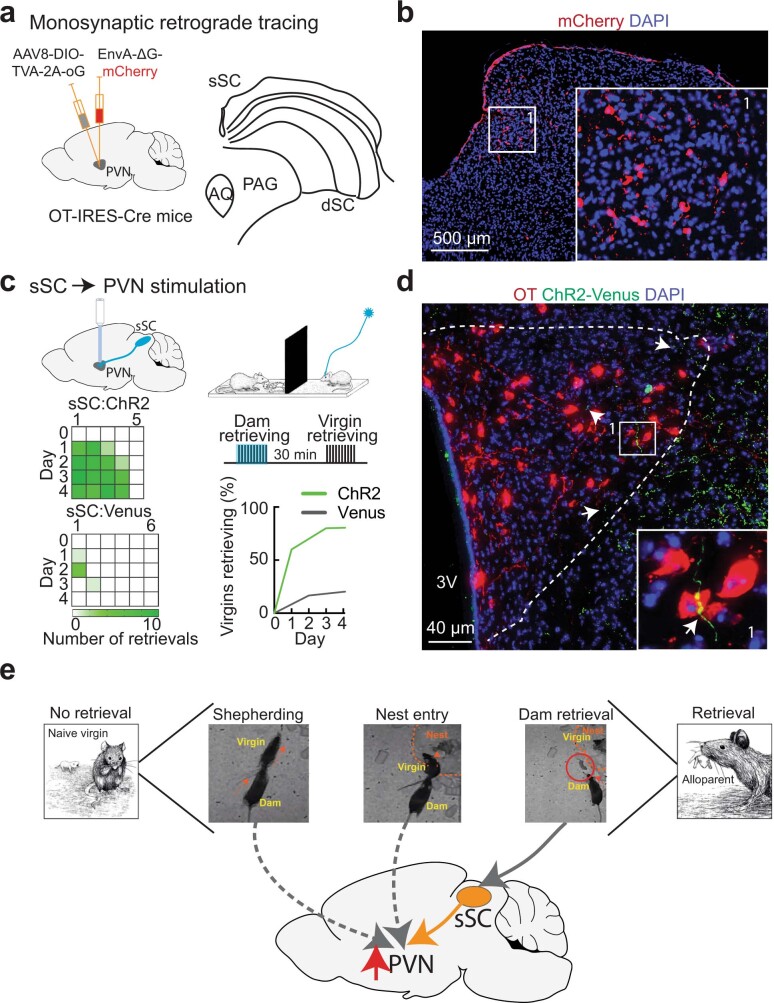
Superior colliculus input to PVN enhances acquisition of pup retrieval. **a**, In *Oxy-IRES-Cre* virgins, we injected an AAV helper virus into PVN, followed two weeks later by injecting a rabies virus that carried mCherry fluorescent protein. Left, retrograde monosynaptic input tracing to OT-PVN neurons. Right, SC anatomy (AQ, aqueduct; PAG, periaqueductal gray; dSC, deep SC layers). **b**, We examined visual structures for mCherry expression (areas providing direct projections to OT-PVN cells). Immunohistochemistry showing mCherry+ cells (red) in the medial superficial layers of ipsilateral SC monosynaptically projecting to OT-PVN neurons. Blue, DAPI stain for cell number. Replicates from N=2 animals. **c**, Top, schematic of experimental design for optogenetic stimulation in PVN of fibres from ipsilateral sSC expressing ‘ChR2-Venus’ or ‘Venus’, during the ‘opaque barrier’ condition (‘Dam retrieving’ and ‘Virgin retrieving’, 10 pup retrieval trials per session; blue, optogenetic stimulation occurring during dam retrieval). Bottom left, individual performance on pup retrieval testing after each observation session. Bottom right, summary of behavioural data (Kaplan-Meier). **d**, Immunohistochemistry showing fibres from sSC (green, arrows) innervating PVN including near OT-PVN neurons (insert). Red, oxytocin neurons; green, Venus; blue, DAPI. 3V, third ventricle. Replicates from N=3 animals. **e**, Schematic of overall hypothesis connecting behavioural interactions between dam and virgin during co-housing to activation of PVN and OT-PVN neurons (red arrow).

We performed monosynaptic rabies-based retrograde tracing to identify visual inputs onto oxytocin neurons, and found projections from medial superficial layers of ipsilateral superior colliculus (sSC→PVN; Extended Data Fig. 9a, b), involved in visual processing^18^. Optogenetic sSC→PVN stimulation could replace visual experience in non-co-housed virgins exposed to maternal retrievals behind an opaque barrier (ChR2-Venus: 4 out of 5 virgins retrieving; control: 1 out of 6 virgins retrieving; *P* = 0.03, Mantel–Cox test; Extended Data Fig. 9c, d). Interactions between virgins and dams or pups in the nest might recruit somatosensory or arousal-based PVN inputs, whereas display of maternal behaviour by dams may lead to collicular visual responses. These results suggest a circuit model by which sensory inputs from social experience converge to activate oxytocin neurons and possibly other hypothalamic cell types to enable parental care (Extended Data Fig. 9e).

Retrievals by dams activated PVN neurons in non-co-housed virgins during observational testing, even though the virgins were not interacting with the dam or pups (Fig. 3h–j). Several of the same cells active during observation also fired during subsequent performance of pup retrieval (Fig. 3h, i; left, 15 out of 65 PVN neurons increased activity during dam retrieval; right, 15 out of 41 of these units increased activity during self-performance). Increased PVN activity was higher on first day versus the last day of observation (Fig. 3j, left), and was elevated only in those non-co-housed virgins that eventually began retrieving (Fig. 3j, right; *n* = 3 learners, 5 non-learners; *P* = 0.0001). Thus, there is a shared hypothalamic cell population that is sensitive to both watching and performing retrieval, reminiscent of mirror or ‘social’ cells in other brain structures^19,20^. This suggests that maternal behaviours performed by the dam are socially transmitted to other females, sometimes visually, via recruitment of virgin PVN neurons including oxytocin cells.

## PVN activity and cortical plasticity

One important neural adaptation with motherhood occurs in the left auditory cortex, which is modulated by oxytocin to amplify responses to pup distress calls to promote retrieval^15,21^. Our results show that although virgin PVN did not respond directly to pup calls, virgin PVN and OT-PVN neurons were recruited by specific experiences during co-housing when abandoned pups would emit distress calls (Extended Data Fig. 6a, b). This might naturally pair distress calls with PVN activation and oxytocin signalling in target brain areas of virgin mice, including regions important for processing vocalizations such as left auditory cortex.

**Fig. 4:**
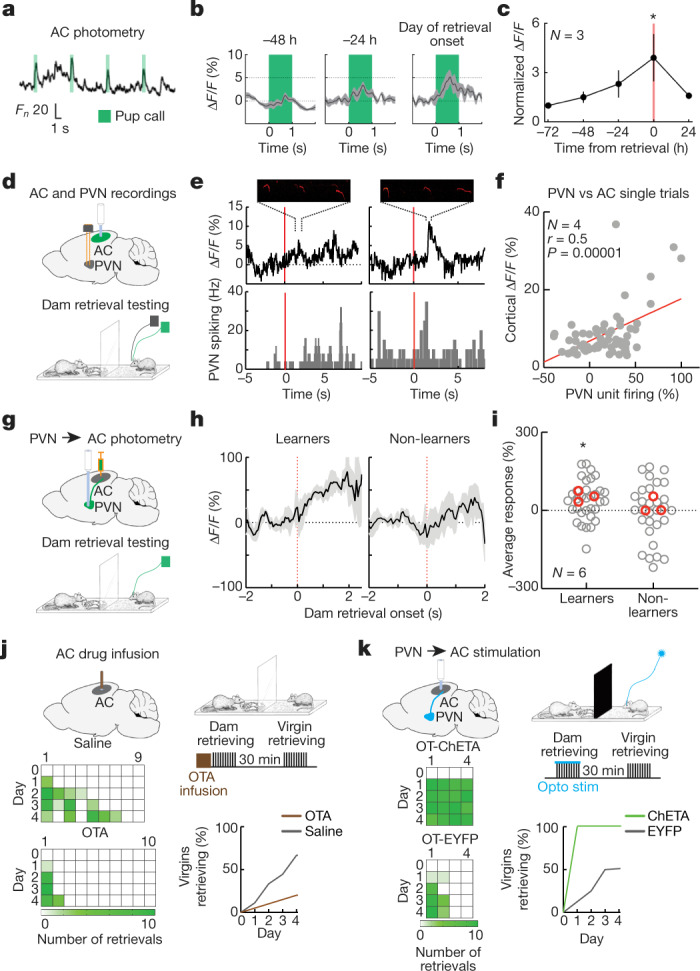
PVN modulation and plasticity of left auditory cortex. **a**, Example photometry recording from virgin female left auditory cortex (AC). **b**, Change in photometry signal during co-housing in example animal. **c**, Summary of photometry data in AC.  **d**, Schematic of simultaneous auditory cortex photometry and PVN single-unit recording during retrieval observation with a transparent barrier. **e**, Example single-trial auditory cortex photometry (insets, pup vocalizations) and PVN spiking before (left) and after (right) virgin began retrieving. Time is zero at dam pup retrieval. **f**, Summary of single-trial responses during dam retrievals. **g**, Schematic of photometry from virgin PVN projections to left AC during transparent-barrier observations. **h**, Photometric responses of auditory cortex from virgin females during observation. Time is zero at dam pup retrieval. **i**, Single-trial (grey) and per-animal responses (red). **j**, Effect of OTA or saline infusion in non-co-housed virgin left auditory cortex before dam retrieval observation. **k**, Optogenetic stimulation (opto stim) of left auditory cortex OT-ChETA fibres during opaque-barrier testing increased virgin retrieval. Data are mean ± s.e.m.; *P < 0.05.

**Extended Data Fig. 10:**
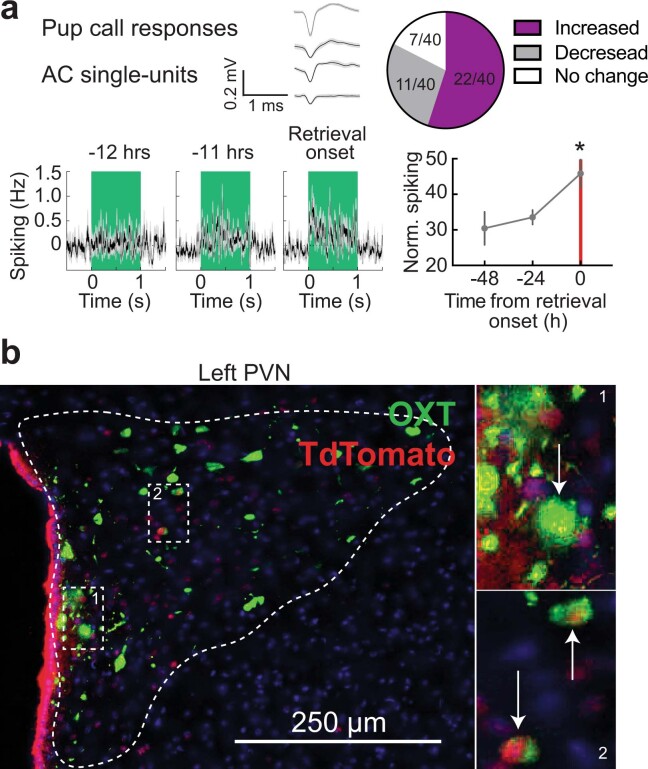
Oxytocinergic modulation from PVN promotes plasticity of AC pup call responses. **a**, Emergence of pup call responses in left auditory cortex preceded retrieval onset; from 71 units recorded in five animals, 40 units in two animals were maintained throughout co-housing (22/40 units significantly increased activity, 11/40 units significantly decreased activity, mean single-unit response across all 40 units to best pup call 48 h before retrieval onset: 30.4±4.6% increase relative to baseline, spiking 24 h before retrieval: 33.5±2.0% increase, spiking on day of first retrieval: 45.8±3.9% increase, p=0.009, one-way ANOVA, n=40 units). Gray shading on unit waveforms represents SEM. **b**, Immunostained tissue section of PVN showing co-labeling of oxytocin peptide (green) and GCaMP6s expressed together with TdTomato (red). Replicates from N=3 animals.

To examine how virgin PVN activation relates to cortical plasticity and eventual pup retrieval, we took recordings from virgin left auditory cortex and PVN using single-unit recordings and fibre photometry. One group of virgins was implanted with tetrodes in left auditory cortex, co-housed with experienced dams and pups for four days, and tested for retrieval on each day. As expected, single-unit responses to played-back pup calls increased during co-housing up to retrieval onset for each mouse (Extended Data Fig. 10a). Similar results were obtained in the second group of wild-type co-housed virgins, with GCaMP6s expression and fibre photometry in left auditory cortex (Fig. 4a–c). We found a progressive increase in photometric signal before the day of retrieval onset (Fig. 4b, c; *P* = 0.003, *n* = 3). Decreased responses 24 h after retrieval onset might reflect further refinement of cortical activity, perhaps via homeostatic mechanisms or inhibitory plasticity^21^, to prevent over-excitability once call-sensitive ensembles have formed.

We next tested how virgin PVN activity related to emergence of cortical pup call responses in non-co-housed virgins. We took advantage of the trial-based structure of retrieval testing and observational testing, and performed simultaneous single-unit recordings from left PVN with photometry from left auditory cortex, together with audio recordings of pup vocalizations during dam pup retrieval (Fig. 4d–f). On some trials during observational testing, virgin PVN spiking transiently increased concomitantly with enhanced cortical responses to pup calls (Fig. 4e). Even before virgins began retrieving pups, single-trial PVN activity correlated with pup call-evoked responses in cortex (Fig. 4f; *r* = 0.5, *P* = 0.00001, *n* = 4). We hypothesize that several episodes of this natural pup call pairing could persistently enhance cortical pup call responses. In this way, the newly parental auditory cortex becomes much more sensitive to pup distress, allowing emergence of rapid behavioural responses.

We tested whether onset of pup retrieval required signalling from virgin PVN to left auditory cortex, performing photometry of PVN→auditory cortex projections during observation of dam retrieval through the transparent barrier (Fig. 4g–i; Extended Data Fig. 10b). In virgins that began retrieving, PVN→auditory cortex projections were activated during observation of dam retrieval (Fig. 4h, i, learners, *P* = 0.02). By contrast, PVN→auditory cortex projections were not significantly activated in virgins that did not learn after observation (Fig. 4h, i, non-learners, *P* = 0.5).

Finally, we investigated whether PVN→auditory cortex input involved oxytocin signalling. Infusing a selective oxytocin receptor antagonist (OTA) into virgin left auditory cortex before transparent-barrier observational testing prevented virgins from learning to retrieve (Fig. 4j, saline, 7 out of 9 virgins retrieving; OTA, 2 out of 10 virgins retrieving; *P* = 0.03). Optogenetic stimulation of OT-PVN→auditory cortex projections during observational testing with the opaque barrier enhanced retrieval learning (Fig. 4k, ChETA-EYFP, 4 out of 4 virgins; EYFP, 2 out of 4 virgins; Mantel–Cox test, *P* = 0.008).

## Discussion

Most parental behaviours are likely to result from both innate and acquired components owing to their rapid onset, reliable performance and importance for species survival^2,4,22–26^. Here we examined how mouse alloparenting behaviour might be acquired by social transmission. We constructed an integrated system that combined continuous days-long videography with audio and neural activity monitoring, for recording documentary videos and subsequent behavioural discovery. We found that experienced mothers shepherd virgins towards pups in the nest but not an empty nest, ensuring that pups are protected by at least one co-caregiver. Virgins could also observe dams retrieving pups after displacement from the nest. This social learning depended on visual input, and we identified direct projections from visual layers of superior colliculus onto PVN oxytocin neurons. Oxytocin may act to amplify neural representations of pup cues such as distress calls in areas important for learned aspects of maternal care, including left auditory cortex. These valuable alloparenting experiences acquired during co-housing are likely to improve the quality of early maternal care when the virgins themselves have litters, and could occur throughout early life in animals living communally in the wild. Socially transmitted parental behaviour would then be useful for helping to ensure the survival and success of offspring in consociating species such as rodents and humans.

## Methods

All procedures were approved under NYU School of Medicine IACUC protocols.

### Data reporting

No statistical methods were used to predetermine sample size. The experiments were not randomized. The behavioural raters were blinded to allocation during co-housing and outcome assessment.

### Behaviour

#### Co-housing

Pup-naive C57Bl/6 virgin female mice were bred and raised at NYU School of Medicine and kept isolated from dams and pups until used for these studies when approximately eight weeks old. For experiments where viral injections were performed, we first allowed two weeks for viral expression before animals were used in experiments. Dams were initially pre-screened to ensure they behaved maternally, meaning that they retrieved pups and built nests; about 1% of dams did not retrieve pups and these animals were not used for co-housing. Naive virgins were initially pre-screened for retrieval or pup mauling before co-housing; around 5% of the naive virgins retrieved at least one pup or mauled pups during pre-screening and these mice were excluded from subsequent behavioural studies.

Co-housing of a virgin female with a mother and litter was conducted for 4–6 consecutive days in 80 × 40 × 50 cm plastic home cages. The floor was covered with abundant bedding material, food pellets and a pack of hydrogel for hydration placed in a corner of the bin and refreshed daily. Nesting material was also placed in the cage. We first placed the dam and her postnatal day 1 (P1) litter in the cage. After the dam was acclimatized for ~30 min, we introduced the virgin female with a tail mark for identification. Well-being of the adult mice and pups was monitored at least twice a day. A surveillance infrared camera system (Blackrock Microsystems) was positioned ~100 cm above the home cage to capture the entire surface. An ultrasonic microphone (Avisoft) was placed in the corner of the cage, ~10 cm above the nest. Two initial cages had a second camera placed on the side but these videos were not analysed for these experiments. For studies of spontaneous pup retrieval by dams and the influence of co-housing, some dams were singly housed with their litter but not with other adults.

In cases where co-housing was done only between a virgin female and pups (Fig. 1c), the pups were returned to the donor mother every 12 h (for at least 48 h) and immediately replaced with new pups. This was done to ensure that they stay alive and healthy despite not being fed during co-housing with the virgin. The procedure was repeated throughout the duration of the co-housing^27^.

#### Pup retrieval testing

This test was used for the initial screening of dams and virgin female mice. In addition, outside of the spontaneous home cage behaviours, we specifically monitored pup retrieval every 24 h by the virgin females. We placed the female mouse to be tested in a behavioural arena (38 × 30 × 15 cm) containing bedding and nesting material; the female was alone, without contact with other animals. Each animal was given 20 min to acclimatize before each testing session began. The entire litter (ranging from 3 to 7 P1–4 pups) were grouped in a corner of the arena and covered with nesting material, and the adult female given an additional 2 min of acclimatization (pup group size did not affect retrieval behaviour; Extended Data Fig. 2c). One pup was removed from the nest and placed in an opposite corner of the arena. The experimental female was given 2 min per trial to retrieve the displaced pup and return it back to the nest; if the displaced pup was not retrieved within 2 min, the pup was returned to the nest and the trial was scored as a failure. If the pup was successfully retrieved, the time to retrieval was recorded and the trial was scored as a success. Another pup was then taken out of the nest, placed away from the nest (varying the position of the isolated pup relative to the nest from trial to trial), and the next trial was begun. After ten trials, pups were placed back into their home cage with their dam. We used an ultrasonic microphone (Avisoft) to verify that isolated pups vocalized during testing.

We reported probability of retrieving out of ten trials. Reliable retrieval was defined as having at least two out of ten successful trials. We used two-way ANOVA and Sidak’s multiple-comparison test corrections to compare probability of retrieving in each group over days, and Student’s *t*-test to compare the day of retrieval onset for each group.

#### Video and audio analysis

Video and audio recordings were synchronized with the neuronal recordings, and then analysed with Adobe Audition and Avisoft. For video recordings we used the BORIS suite for scoring of behavioural observations. Three separate teams of independent scorers (two scorers from the Sullivan laboratory, three scorers from the Carcea laboratory and four scorers from the Froemke laboratory) were trained in a similar way on how to identify relevant individual and social behaviours during co-housing, and then scored the videos blind to the conditions. The results from each raster were compared and compiled, and results from each lab were cross-validated. Nest entry was considered the moment when the head of the animal entered the nest. Nest exit was considered the time when the rear of the animal left the nest. We used two-way ANOVA and Sidak’s multiple-comparison test to compare pup retrieval rates and time in nest across days for each group.

Any event in which the dam chased the virgin towards the nest was identified as a shepherding event (that is, where distance from start to nest was greater than distance from end to nest). To determine the distance from nest during shepherding, we measured the distance from the bottom left corner of the cage to the position of the snout of the mouse, and to the position of the nest center. We then calculated distance from the virgin to nest. In cases of physical contact, start of shepherding was considered to be the moment when the dam made contact with the virgin, and the end of shepherding was the moment when the virgin stopped running. In some cases (especially later into co-housing), we noticed that virgins started running as soon as they noticed the dam approaching; in those cases, the start of shepherding was considered to be the moment when the virgins started running after the dam’s approach. For Fig. 1i, we used paired *t*-tests to compare distance from start of shepherding to nest with the distance from end of shepherding to nest. For Fig. 1j, we used one-sample Student’s *t*-tests to determine if the daily frequency of shepherding was higher than 0.2 events per h (which was the average rate of dam–virgin chases in absence of pups). Audio recordings were processed in Adobe Audition, and isolation or distress calls were distinguished from adult calls and wriggling calls on the basis of the characteristic statistics (bout rate of 4–8 Hz and frequencies of 40–90 kHz).

#### Observation of experienced retrievers

We first confirmed that virgins did not retrieve and dams retrieved at 100% at baseline. The exposures were done in standard behavioural arena (38 × 30 × 15 cm). The virgin and dam were acclimatized for 20 min, then the nest with pups was transferred to this arena. After another 5–10 min, we manually isolated one pup at a time so that the dam would retrieve the pup back into the nest. We repeated this for ten times per session. In the experiments where either a transparent or an opaque divided the cage, the two adult animals were acclimatized on opposite sides of the barriers. After exposure, the adult animals were separated and the virgins were tested for pup retrieval 30 min later, as described above. As the preparation for testing and the acclimatization to the testing cage also took 30 min, this amounted to a total 60-min interval between virgin observation and testing of responses to isolated pups. The exposure was repeated for four sessions (one per day). A virgin that retrieved at least once during the four days of observation was considered as having acquired pup retrieval behaviour. We used chi-square exact tests to compare retrieval between conditions: wild-type mice with no barrier, wild-type mice with transparent barrier, wild-type mice with opaque barrier, and OXTR-KO virgins with transparent barrier.

### Surgery

#### Viral injections

To test the effects of DREADDs, stereotaxic viral injections were performed in *Oxy-IRES-Cre* mice^15^. Mice were anaesthetized with 0.7–2.5% isoflurane (adjusted on the basis of scored reflexes and breathing rate during surgery), placed into a stereotaxic apparatus (Kopf), and bilateral craniotomies performed over PVN (from bregma: 0.72 mm posterior, 0.12 mm lateral). Injections were performed at a depth of 5.0 mm with a 5 µl Hamilton syringe and a 33 gauge needle. Cre-inducible AAV2 hSyn::DIO-hM4D(Gi)-mCherry (University of North Carolina Viral Core) virus was injected into PVN at 0.1 µl min^−1^ for a final injection volume of 1.2–1.5 µl. The craniotomy was sealed with a silicone elastomer (World Precision Instruments), the skin sutured. Animals were used for experiments after two weeks to allow for viral expression.

For monosynaptic retrograde tracing of inputs on OT-PVN neurons, we injected 0.5 µl of a helper virus, AAV8-DIO-TVA-2A-oG (Salk Institute), in the left PVN of *Oxy-IRES-Cre mice*. Two weeks later, we injected 0.2 µl of a pseudotyped rabies virus, EnvA G-deleted rabies-mCherry (Salk Institute) at the same location. After another two weeks, we perfused the animals and collected the brains for histology.

For optogenetic stimulation of superficial superior colliculus (sSC)→PVN projections, wild-type virgins were injected in the left sSC (from bregma: −3.08 mm posterior, −0.27 mm lateral, −1.9 mm ventral) with 0.5 µl of either AAV9-pACAGW-ChR2-Venus or control AAV9-CAG-Venus virus. We then implanted a 400 µm optic fibre in the left PVN.

For fibre photometry from auditory cortex, we performed viral injections into the left auditory cortex (~1.7 mm anterior from the occipital suture, 0.5 mm lateral from the temporal ridge, 1 mm ventral from pia), using a similar procedure. These coordinates corresponded with the ones previously published from our laboratory^15^: 2.9 posterior, 4.0 lateral (left) from bregma. We also oriented using local markers consisting in the branching patterns of the rhinal vein and of the middle cerebral artery, to target the auditory cortex as previously described^28^. We injected 1 µl of AAV1 Syn::GCaMP6s (Addgene) at a titre of 1 × 10^13^ vg ml^−1^ in the auditory cortex of wild-type mice. Following the injection, we implanted a 400 µm optical fibre (ThorLabs) just above the auditory cortex, or inserted in the superficial layers of the cortex (200–300 µm below the pial surface). For photometry from PVN→auditory cortex projection neurons, we injected 50 nl of AAVrg-hSyn1-GCaMP6s-P2A-nls-dTomato virus (Addgene) at three locations within the left auditory cortex. The virus titre was 1.31 × 10^12^ vg ml^−1^, and we injected at 10 nl min^−1^.

For optogenetically manipulating OT-PVN→auditory cortex projections, we injected the left PVN of Oxy-IRES-Cre virgins with 0.5 µl of either AAV9-Ef1a-DIO-ChETA-EYFP or of AAV9-pCAG-FLEX-EYFP-WPRE. We then implanted a 400-µm optic fibre in the left auditory cortex.

#### Microdrive implantations

For in vivo single-unit electrophysiology we implanted microdrives either in the left PVN (Figs. 2–4) or the left auditory cortex (Fig. 4). We built microdrives using the parts and instructions for 4-tetrode Versadrives (Neuralynx), adapting these instructions for two bundles each made up of eight 12.5-µm Nichrome wires. The day of the implantation, the wires were cleaned and gold plated to achieve impedances <500 kΩ. After the virgin female mice were anaesthetized with isoflurane, a craniotomy (1.5–2 mm in diameter) was performed above the target structure, and two additional small craniotomies were performed in the occipital bone and the right parietal bone for insertion of bone screws. The ground and reference wires of the microdrives were soldered separately to these two bone screws. The dura was removed at the desired implantation site and the electrode bundles were slowly lowered to ~500 µm above the target brain structure (4 mm ventral from pia for PVN). For recordings from the auditory cortex, we first acutely recorded multiunit activity with tungsten electrodes to localize auditory cortex during implantation procedure. Auditory cortex was identified with pure tones (60 dB SPL, 7–79 kHz, 50 ms, 1 ms cosine on–off ramps) delivered in pseudo-random sequence at 0.5–1 Hz. The craniotomy was covered with mineral oil and silicone elastomer, and the microdrive was secured to the skull using dental cement (C&B Metabond).

For optically identified recordings from OT-PVN units, we used *Oxt-IRES-Cre* mice bred into a C57BL/6 background. Prior to the microdrive implantation, we injected the PVN of these animals with 1–2 µl of AAV1-CAG::DIO-ChR2 at a titre of 1 × 10^13^ vg ml^−1^ (in one mouse, we injected AAV2-EEF1::DIO-ChETA at a titre of 1 × 10^13^ vg ml^−1^). Then, after implanting and cementing the microdrive, we rotated the head of the animal at a 45º−50º angle around the anterior–posterior axis, and another craniotomy was done at 0.72 mm posterior and 3.5 mm left from bregma. A 400 µm optical fibre for delivering blue light was slowly lowered at this position for 4.5 mm below the brain surface. For dual recordings, we implanted a microdrive in left PVN, injected the GCaMP virus (as described above) and implanted an optic fibre in the left auditory cortex during the same surgery.

#### Optical fibre implantation

For fibre photometry we used 400-μm-diameter 0.48NA optic fibres inserted into 2.5-mm-diameter ceramic ferrules (Doric Lenses). For recordings of PVN→auditory cortex, we used 1.25-mm metal ferrules, with fibres 1 mm long (below ferrule) for recordings in the auditory cortex and 5 mm long for recordings in PVN. They were implanted during the same surgery as the viral injections, and cemented to the skull as described above.

### Recordings

#### Single-unit recordings during behaviour

Neuronal recordings were performed at 30 kHz sampling rate using the Cereplex µ headstage, a digital hub and a neuronal signal processor (Blackrock Microsystems). The recordings were synchronized with a video recording system (Neuromotive, Blackrock). Before the start of the experiment the electrode bundles were lowered into the target structure and then advanced daily by ~70 µm. For PVN recordings, optical tagging tests were performed at the end of each co-housing day prior to lowering the electrodes for unit identification on the following day. Optical identification was performed in a separate clean cage in absence of pups or dam. The optical fibre was connected to a blue laser triggered using an analogue output from the recording system. The laser pulses were 5 ms in duration and delivered at either 2 or 5 Hz. Light intensity was controlled by adjusting the output of the laser, and at least three different intensities were tested in each mouse. The optical identification procedure was <5 min each day, and we used lower frequency (2 Hz) stimulation to minimize the likelihood of functional changes induced by optogenetic stimulation. To allow free movement of the implanted mice, we used a pulley combined with a fibre-optic and electrical rotary joint–commutator (Doric Lenses).

#### Spike sorting

Neurons were isolated offline using Cerebrus and BOSS software (Blackrock Microsystems), as previously described^29^. A notch filter was applied to eliminate the line noise, then a high-pass filter of 250 Hz was applied, and a threshold multiplier was used to extract spike from signal energy between 1–5 kHz. We then used principal component analysis to extract spike features and to manually cluster them in different units. We eliminated spikes that violated a refractory period of 2–3 ms, and spikes that were simultaneously recorded in 10 or more channels (as potential artifacts). For tetrode recordings, we used a similar procedure in BOSS (Blackrock) that allowed triangulation of waveforms from the four tetrode channels. For identifying oxytocin neurons in optically tagged recordings, we aligned neuronal activity to the onset of the blue light pulse^30^. Neurons that reliably fired (≥70% of trials) at short latency (≤4 ms) after the onset of blue light were selected as being oxytocin neurons.

#### Spike train analysis

To analyse changes in firing patterns, we aligned the spike trains to the onset of the behavioural episode and calculated the firing rate for the duration of the behaviour. In the case of ‘nest entry’, we used a 40-s cut-off, in order to minimize the impact from other behaviours that occur in the nest over longer time periods (for example, grooming, sleeping and nest building); this cut-off was chosen as most nest dwell times were ≤40 s (Extended Data Fig. 7b). For each behavioural episode we also calculated the corresponding baseline firing rate, either for an interval similar in duration to the behavioural episode (in the case of nest entry), or for 10 s before the start of the behavioural episode (for shepherding and dam retrievals). The change in firing rate was calculated as: (Spiking_behaviour_ − Spiking_baseline_) × 100/(Spiking_behaviour_ + Spiking_baseline_), where Spiking_behaviour_ was firing rate during the behavioural event, and Spiking_baseline_ was firing rate during baseline. To determine whether these changes in firing rate were statistically significant, we shifted the behaviour intervals by a random value between −500 and 500 s, and then calculated new firing rates and modulation percentages for 1,000 random shifts. All cells for which the modulation of activity in the unshifted data was greater than modulation in 950 shuffles were considered to be potentially significantly activated^31^. Cells for which the change in firing rate was smaller than the change in 950 shuffles were considered to be potentially significantly suppressed. For each unit, we computed the *P*-value from the permutation analysis and then applied a false-discovery rate correction for the multiple comparisons performed on each data subset (all OT-PVN or all PVN neurons recorded in a day), to identify neurons with significant changes in firing rate. The threshold for *q*-value was set 0.05, that is, 5% accepted false-discovery rate. To determine if the fraction of activated neurons is meaningful, we then applied a one-sided Fisher’s exact test (with the null hypothesis of zero neurons being activated) with the threshold for alpha set at 0.05 (ref. ^32^). To compare fractions of OT-PVN and PVN neurons activated by a specific behaviour, we applied a two-sided Fisher’s exact test, and calculated the effect size as the relative risk: the probability of neurons being activated in the OT-PVN group/the probability of neurons being activated in the PVN group, using the Koopman asymptotic score for calculating 95% confidence intervals. To investigate how PVN activity correlated with activity in the auditory cortex, a population average change in PVN firing was calculated for each maternal-retrieval trial of the observational learning procedure.

To quantify unit activity during behavioural episodes, we aligned the neuronal data to the onset of the behaviour, and binned the spiking data in 250-ms bins for an interval between −20 s and +40 s (−10 s to +20 s in the case of shepherding episodes). We then calculated the *z*-score value for each bin in the chosen time window; for display purposes, we used bounds of −3 (minimum *z*-score) and +3 (maximum z-score). For responses to pup call playback, spike trains from single-unit recordings in PVN or auditory cortex were aligned to the onset of the played-back vocalization. The average firing rate during the pup call (1-s interval) was normalized to the firing rate during baseline (1 s preceding pup call onset).

To measure the cross-correlation for simultaneously recorded OT-PVN and PVN single-units during co-housing (Extended Data Fig. 7d, e), we calculated the normalized zero-lag correlation of spike counts (in 10-ms bins) across all pairs of units^33^, separately for each of the three behavioural conditions (shepherding, nest entry and maternal retrievals). We then averaged *z*-transformed *r* values across all events of each behaviour, to obtain a single cross-correlation value for each pair and each behaviour. Higher correlation *r* values reflect higher synchrony within the pair.

#### Fibre photometry

To perform fibre photometry, we connected the optical fibre implanted in the auditory cortex and housed in a ceramic ferrule to a custom-built photometry rig^34^. A 400-Hz sinusoidal blue light was delivered via the optical fibre from an LED (30 µW) for GCaMP6s excitation. We collected the emitted (green) light via the same optical fibre, used a dichroic mirror and appropriate filters to direct emitted light to a femtowatt silicon photoreceiver (Newport), and recorded using a real-time processor (RX8, TDT). The analogue readout was then low-pass filtered at 20 Hz. The intensity of blue light excitation was adjusted to produce similar baseline fluorescence levels across sessions in the same mouse. The sound processor used for delivering the pup calls (RZ6, TDT) was synchronized with the fibre photometry system.

For investigating changes in neuronal activity of the left auditory cortex throughout co-housing, we recorded responses to six different played-back pup calls every 24 h. To avoid possible changes in the magnitude of calcium transients with changes in the position of the mouse relative to the speaker, in some recordings we restrained the mouse in a mash cup, at the same distance from the speaker. Data from restrained or unrestrained mice showed similar trends, and were thus pooled together. To determine the evoked response recorded with fibre photometry, we calculated the Δ*F*/*F* for each pup call interval.

### Perturbations

#### Chemogenetic suppression of oxytocin neurons during co-housing

*Oxy-IRES-Cre* virgin female mice expressing in OT-PVN cells either DREADD coupled to inhibitory G-protein (DREADDi, also known as hM4D(Gi)) and fused with mCherry, or mCherry alone (as control), were co-housed with mothers and pups as described above. In both experimental (*Oxy-IRES-Cre* virgins expressing DREADDi-mCherry) and control (*Oxy-IRES-Cre* virgins expressing mCherry) cohorts, clozapine-*N*-oxide (CNO) was administered in the drinking water at a concentration of 25 mg l^−1^ (ref. ^35^). Five sugar pellets were added in each bottle to obscure the taste of CNO. At this concentration, for the average mouse body weight (~30 g) and average daily water consumption in mice (~6 ml), the average CNO consumption was ~5 mg kg^−1^ per day.

Three out of seven mice in the mCherry^+^ CNO cohort had DREADDi virus expressed outside of the PVN due to mis-targeting. These mice were also treated with CNO; as their behaviour was similar to the mCherry-expressing mice treated with CNO—we combined data from these groups together in Fig. 2a. For each animal, the retrieval probability for the first two days of co-housing was averaged. For Extended Data Fig. 4b, retrieval testing was done in animals that stably retrieved at baseline and as such were not subjected to the co-housing procedure.

#### Optogenetic stimulation

For optogenetics, we connected the optical fibre implanted either in PVN or in the left auditory cortex of the virgin mouse to a blue light laser (OptoEngine). Light pulses (5 ms) were delivered at 20 Hz (for Extended Data Fig 9; ref. ^21^) or 30 Hz (for Fig. 4; ref. ^15^), while the dam performed 10 retrieval trials across an opaque barrier from the virgin. To minimize potential adaptation or synaptic depression, stimulation was performed for ≤5 min, enough time for dams to complete 10 retrievals. After 30 min, the non-co-housed virgin was tested for retrieval. This procedure was repeated once a day for four consecutive days.

#### Drug infusion

To determine the role of cortical oxytocin receptor activation during observation of pup retrieval, we implanted cannulas in the left auditory cortex of naive virgin females^15^. After mice recovered from surgery, we placed them in the observational chamber, and then infused either saline or the oxytocin receptor antagonist OTA (1 µM solution in saline) through the cannula. We injected a volume of 1.5 µl at a rate of 1 µl min^−1^. We removed the internal from the cannula five minutes after the end of injection to allow the solution to diffuse, and then allowed the virgins to acclimatize to the observational chamber for another 10 min. After this, we exposed virgins to maternal retrievals across the transparent barrier as described above. Drug conditions were compared with a survival log-rank analysis to determine effects on retrieval onset across groups.

### Immunohistology

To verify viral expression at the end of the experiments, animals were fixed with transcardiac perfusions of 4% paraformaldehyde. Brains were removed and further preserved in a paraformaldehyde solution for 1–2 h at 4 °C. Afterwards, brains were sequentially cryopreserved in 15% and then 30% sucrose solution, embedded in OCT solution and sectioned (30 µm) with a cryostat then mounted on positively charged slides. Immunohistochemistry was performed on the mounted sections as previously described^29,36^. Sections were blocked in 5% goat serum solution for 1 h at room temperature or for 14 h at 4 °C. A solution of the appropriate primary antibodies was diluted in 1% goat serum and 0.01% Triton X solution and then applied for 24 h at 4 °C. We used a rabbit anti-oxytocin antibody (EMD Millipore, 1:500), mouse anti-oxytocin antibody (a gift from H. Gainer at national Institutes for Health), a chicken anti-GFP (Aves, 1:500) and a chicken anti-mCherry (Abcam, 1:1,000) antibody. The sections were washed in PBS solution, and a solution of fluorophore-conjugated secondary antibodies applied for 1.5 h at room temperature. All secondary antibodies were from Jackson Immunoresearch and used at 1:200. Slides were examined and imaged using a Carl Zeiss LSM 700 confocal microscope with four solid-state lasers (405/444, 488, 555 and 639 nm) and appropriate filter sets. For imaging sections co-stained with multiple antibodies, we used short-pass 555 nm (Alexa Fluor 488), short-pass 640 nm (Alexa Fluor 555), and long-pass 640 nm (Alexa Fluor 647) photomultiplier tubes.

### Micro-computed tomography imaging

The localization of the implanted electrodes was assessed in vivo using μCT scans in post-implanted mice, followed by co-registration with an online digital MRI mouse brain atlas. The µCT datasets were acquired using the μCT module of a MultiModality hybrid micro-Positron Emission Tomography–µCT Inveon Scanner (Siemens Medical Solutions). The Inveon scanner is equipped with a 165 mm × 165 mm x-ray camera and a variable-focus tungsten anode x-ray source operating with a focal spot size of less than 50 μm. The scan consisted of a 20-min whole-head acquisition over an axial field of view of 22 mm and a transaxial of 88 mm with a resolution of 21.7 μm pixels binned to 43.4 μm. 440 projections were acquired using a 1-mm aluminum filter, a voltage of 80 kV, and a current of 500 μA. The datasets were reconstructed using the Feldkamp algorithm^37^.

The hybrid scanner was equipped with a M2M Biovet (Cleveland) module used to monitor continuously vital signs. All mice were monitored continuously throughout the scanning session via a respiration sensor pad (SIMS Graseby). The imaging scan consisted of initially placing each mouse in an induction chamber using 3–5% isofluorane exposure for 2–3-min until the onset of anesthesia. The mouse was then subsequently positioned laterally along the bed palate over a thermistor heating pad in which 1.0% to 1.5% isofluorane was administered via a 90º angled nose cone throughout the scan. The head of each subject was judiciously oriented perpendicular to the axis of the mouse body so that the extracranial part of the implanted electrode could be easily kept away from the field of view of the µCT image acquisition. Importantly, the large extracranial metal components and dental cement of the implant can cause beam hardening that can appear as cupping, streaks, dark bands or flare in the µCT^38–40^. To this effect, the head positioning helped reduce the risks of image artifacts that could be induced by the implant along the path of the X-ray beam.

Unlike MRI, μ-CT imaging can be performed on subjects with metal implants. However, lack of the soft tissue contrast of the µCT limited its usefulness to provide the needed brain anatomical detail in order to verify the electrode correct localization. Our approach combined registration of post-implant µCT with an existing online MRI brain atlas for adult C57Bl/6 mice from the Mouse Imaging Centre: (https://wiki.mouseimaging.ca/display/MICePub/Mouse+Brain+Atlases).

The three-dimensional MRI mouse brain atlas was established by acquiring 40 individual ex vivo mice using T2-weighted sequence on a 7-Tesla scanner. All the data were averaged and resulted into a 40 µm isotropic resolution dataset detailed in Dorr et al. (ref. ^41^). The hypothalamic PVN was manually segmented and color-coded, with the guidance of the P56 coronal Allen mouse histology brain atlas^42^. This region was set as the target of reference. A rigid co-registration between µCT and the modified MRI atlas images was systematically performed using a commercial software Amira (Thermo Fisher Scientific). Both datasets were then overlaid for visual analysis and to determine the sub-millimetric localization of the electrode tip.

### Reporting summary

Further information on research design is available in the Nature Research Reporting Summary linked to this paper.

## Online content

Any methods, additional references, Nature Research reporting summaries, source data, extended data, supplementary information, acknowledgements, peer review information; details of author contributions and competing interests; and statements of data and code availability are available at 10.1038/s41586-021-03814-7.

## Supplementary information

Supplementary Information This file contains a Supplementary Discussion.

Reporting Summary

Supplementary Video 1 Example of spontaneous pup retrieval by mother occurring during co-housing (same as in Fig. 3a).

Supplementary Video 2 Example of virgin spending time in nest with pups after days of co-housing; same damvirgin dyad recorded on days one through four.

Supplementary Video 3 Retrieval onset in virgin co-housed with dam and pups (‘D+’). Example pup retrieval testing on day 0, day 1, and day 3.

Supplementary Video 4 Retrieval onset in virgin co-housed with pups (‘D-’). Example pup retrieval testing on day 0, day 1, and day 5.

Supplementary Video 5 Example of dam shepherding virgin back to nest (same event as in Fig. 1g).

Supplementary Video 6 Video with more examples of dam shepherding from different co-housing pairs.

Supplementary Video 7 Video of dam shepherding implanted and tethered virgin.

Supplementary Video 8 Video with more examples of self-generated retrievals from different co-housing pairs.

Supplementary Video 9 Video of self-generated retrieval by dam with implanted and tethered virgin in nest.

Supplementary Video 10 Example of dam bringing pup near virgin.

Supplementary Video 11 Testing social transmission of maternal behaviour with transparent barrier separating virgin from dam and pups; after two days, virgin was retrieving.

Supplementary Video 12 Testing social transmission of maternal behaviour with opaque barrier separating virgin from dam and pups; virgin did not begin retrieving even after four days.

## Data Availability

Data and statistical tests are available in the supplementary data sheet provided with this manuscript. The video data generated during this study are available in Databrary (https://nyu.databrary.org/volume/1333/slot/55145/-). Other data are available in Zenodo (https://zenodo.org/record/5039601#.YNvaYEwpCF5) and DANDI (https://gui.dandiarchive.org/#/dandiset/000114). The NYU Data Catalog record will point to the most updated locations of this dataset (https://datacatalog.med.nyu.edu/dataset/10449?tak=160dbb2281acf5). Source data are provided with this paper.

## Source data

Source Data Fig. 1
Source Data Fig. 2
Source Data Fig. 3
Source Data Fig. 4
Source Data Extended Data Fig. 3
Source Data Extended Data Fig. 4
Source Data Extended Data Fig. 6
Source Data Extended Data Fig. 7
Source Data Extended Data Fig. 8
Source Data Extended Data Fig. 9
Source Data Extended Data Fig. 10

## Code availability

The Matlab code is available at github (https://github.com/ic283/Carcea-et-al.-2021).
