# Supplementary material for: Oxytocin neurons enable social transmission of maternal behaviour
Source: Nature. 2021 Aug 11;596(7873):553–7. doi: 10.1038/s41586-021-03814-7 (PMC8387235; doi:10.1038/s41586-021-03814-7)
Supplement: Supplementary file 1 — This file contains a Supplementary Discussion. [file 41586_2021_3814_MOESM1_ESM.pdf]

nature portfolio

https://doi.org/10.1038/s41586-021-03814-7

Supplementary information

Oxytocin neurons enable social transmission of maternal behaviour

In the format provided by the authors and unedited

Supplementary Discussion

It has been difficult to directly connect modifications of neural circuits to changes in behavior[43-45], for at least two reasons: first, a suitable behavioral model is required for measuring or manipulating neural circuits over the entire time-course of behavioral changes; and second, changes in neural activity may occur rapidly and transiently, at various times for different animals. Furthermore, behaviorally-relevant plasticity in vivo generally requires neuromodulation (such as activation of the oxytocin system), combined with local circuit activity driven by sensory experience[46-48].

Our approach here took advantage of the speed and reliability of maternal behavior onset (and pup retrieval in particular), to record directly for the first time from identified oxytocin neurons in non-lactating mice during social interactions. This allowed us to relate episodes of PVN oxytocin neuron firing to moments of modulation and plasticity within left auditory cortex, required for pup retrieval behavior[15,23]. It is important to note that the PVN population might contain non-oxytocin cells sensitive to social interactions[25,49] and possibly non-optically-tagged oxytocinergic neurons, and so here we refer to non-OT-PVN units simply as unidentified 'PVN' units. Units were identified at the end of each day to minimize impact of optogenetic stimulation. Repeated optogenetic stimulation might impact neural circuit function and behavior, although there were no apparent differences we detected in these animals.

It remains to be determined precisely how virgins sense and watch experienced mothers retrieving pups, and what other cues from the dam and pups such as vocalizations, touch, or olfactory signals[47,48,50-52] might help instruct or incentivize the virgin. Other studies of observational learning across species have highlighted the importance of a range of sensory modalities[53-59]. It will also be important to understand how multisensory inputs are integrated for

oxytocin release across different species; e.g., if olfactory cues might be more effective than visual cues in rodents, whereas visual cues might be more essential in humans. Even in the virgin mice receiving optogenetic stimulation behind the opaque barrier, these animals were still exposed to numerous other multisensory cues (both during dam retrieval, as well as when they are tested on pup retrieval). Future studies will be required to determine if artificial activation of specific sets of inputs could suffice to fully replace the social and parental contexts, or which sets of cues are required during observation and during self-performance for effective learning to occur. Additionally, it remains to be determined how this activity might lead to bona fide oxytocin release, enabling subsequent cortical modifications and onset of alloparenting.
