# Supplementary material for: Oxytocin neurons enable social transmission of maternal behaviour
Source: Nature. 2021 Aug 11;596(7873):553–7. doi: 10.1038/s41586-021-03814-7 (PMC8387235; doi:10.1038/s41586-021-03814-7)
Supplement: Supplementary file 2 — Reporting Summary [file 41586_2021_3814_MOESM2_ESM.pdf]

nature research

Corresponding author(s): Robert C. Froemke

Last updated by author(s): Apr 28, 2021

Reporting Summary

Nature Research wishes to improve the reproducibility of the work that we publish. This form provides structure for consistency and transparency in reporting. For further information on Nature Research policies, see our Editorial Policies and the Editorial Policy Checklist.

Statistics

For all statistical analyses, confirm that the following items are present in the figure legend, table legend, main text, or Methods section.

n/a	Confirmed	
☐	☒	The exact sample size (*n*) for each experimental group/condition, given as a discrete number and unit of measurement
☐	☒	A statement on whether measurements were taken from distinct samples or whether the same sample was measured repeatedly
☐	☒	The statistical test(s) used AND whether they are one- or two-sided<br>*Only common tests should be described solely by name; describe more complex techniques in the Methods section.*
☐	☒	A description of all covariates tested
☐	☒	A description of any assumptions or corrections, such as tests of normality and adjustment for multiple comparisons
☐	☒	A full description of the statistical parameters including central tendency (e.g. means) or other basic estimates (e.g. regression coefficient) AND variation (e.g. standard deviation) or associated estimates of uncertainty (e.g. confidence intervals)
☐	☒	For null hypothesis testing, the test statistic (e.g. *F*, *t*, *r*) with confidence intervals, effect sizes, degrees of freedom and *P* value noted<br>*Give P values as exact values whenever suitable.*
☒	☐	For Bayesian analysis, information on the choice of priors and Markov chain Monte Carlo settings
☒	☐	For hierarchical and complex designs, identification of the appropriate level for tests and full reporting of outcomes
☒	☐	Estimates of effect sizes (e.g. Cohen's *d*, Pearson's *r*), indicating how they were calculated

Our web collection on statistics for biologists contains articles on many of the points above.

Software and code

Policy information about availability of computer code

Data collection	Blackrock Central single-unit recording software, Synapse TDT software for acquisition of photometry data, Avisoft software for acquisition of sounds
Data analysis	Matlab custom codes for analysis of single-unit, photometry and behavior data, Boris for behavioral analysis

For manuscripts utilizing custom algorithms or software that are central to the research but not yet described in published literature, software must be made available to editors and reviewers. We strongly encourage code deposition in a community repository (e.g. GitHub). See the Nature Research guidelines for submitting code & software for further information.

Data

Policy information about availability of data

All manuscripts must include a data availability statement. This statement should provide the following information, where applicable:

- Accession codes, unique identifiers, or web links for publicly available datasets
- A list of figures that have associated raw data
- A description of any restrictions on data availability

Provide your data availability statement here.

Field-specific reporting

Please select the one below that is the best fit for your research. If you are not sure, read the appropriate sections before making your selection.

☒ Life sciences ☐ Behavioural & social sciences ☐ Ecological, evolutionary & environmental sciences

For a reference copy of the document with all sections, see nature.com/documents/nr-reporting-summary-flat.pdf

Life sciences study design

All studies must disclose on these points even when the disclosure is negative.

Sample size	Sample size was determined using statistical power analysis, with alpha set at 0.05
Data exclusions	No data was excluded
Replication	Behavioral scoring was performed by several different observers, and randomly cross-checked to insure replicability (DR, MO). Analysis of neuronal data was performed separately by two scientist (IC and JSR), and cross-validated. Chemogenetic experiments, observational learning experiments, OTA infusion experiments were performed separately by different scientists (IC, RO, NLC, HL, MAT, JMN), and at two different institutes (NYU and Rutgers NJMS).
Randomization	Animals were randomly assigned to experimental conditions.
Blinding	When possible for behavioral and immunohistochemistry analysis, observers were blinded to conditions.

Reporting for specific materials, systems and methods

We require information from authors about some types of materials, experimental systems and methods used in many studies. Here, indicate whether each material, system or method listed is relevant to your study. If you are not sure if a list item applies to your research, read the appropriate section before selecting a response.

Materials & experimental systems

n/a	Involved in the study	
☐	☒	Antibodies
☒	☐	Eukaryotic cell lines
☒	☐	Palaeontology and archaeology
☐	☒	Animals and other organisms
☒	☐	Human research participants
☒	☐	Clinical data
☒	☐	Dual use research of concern

Methods

n/a	Involved in the study	
☒	☐	ChIP-seq
☒	☐	Flow cytometry
☒	☐	MRI-based neuroimaging

Antibodies

Antibodies used	Primary antibodies: 1. rabbit anti-oxytocin antibody (EMD Millipore, 1:500), 2. mouse anti-oxytocin antibody (a gift from Dr. Harold Gainer at NIH), 3. a chicken anti-GFP (Aves, 1:500) and 4. a chicken anti-mCherry (Abcam, 1:1000). Secondary antibodies: All secondary antibodies were from Jacksons Immunoresearch
Validation	Primary antibodies:<br>1 & 2 anti-oxytocin antibodies have been validated in the Carcea lab at Rutgers BHI by showing over 90% co-localization with EYFP staining in PVN of Oxy-Cre;Ai32 mice (expressing ChR2-EYFP in oxytocin neurons). Relevant citations: Tsuda et al., NeuroReport 2011;<br>3. This antibody was validated by manufacturer as stated on their website: 'Antibodies were analyzed by western blot analysis (1:5000 dilution) and immunohistochemistry (1:500 dilution) using transgenic mice expressing the GFP gene product.'<br>4. This antibody was validated by manufacturer as stated on their website: 'Positive control: Lysate of HEK293 cells transfected with pFin-EF1-mCherry vector; HEK293 cells transfected with pFin-EF1-mCherry vector.'<br><br>Secondary antibodies:<br>For all secondary antibodies, we performed 'secondary only' controls.

Animals and other organisms

Policy information about studies involving animals; ARRIVE guidelines recommended for reporting animal research

Laboratory animals	Mice, C57/B6, females at 8 weeks old (virgins) and 10 weeks old (dams)

Wild animals	*Provide details on animals observed in or captured in the field; report species, sex and age where possible. Describe how animals were caught and transported and what happened to captive animals after the study (if killed, explain why and describe method; if released, say where and when) OR state that the study did not involve wild animals.*
Field-collected samples	*For laboratory work with field-collected samples, describe all relevant parameters such as housing, maintenance, temperature, photoperiod and end-of-experiment protocol OR state that the study did not involve samples collected from the field.*
Ethics oversight	All procedures were approved under NYU School of Medicine IACUC committee and by Rutgers NJMS IACUC committee.

Note that full information on the approval of the study protocol must also be provided in the manuscript.
